# Endoscopy Lifetime Systems Architecture: Scoping Out the Past to Diagnose the Future Technology

**DOI:** 10.3390/systems10050189

**Published:** 2022-10-14

**Authors:** Craig M. Browning, Robert Cloutier, Thomas C. Rich, Silas J. Leavesley

**Affiliations:** 1 Department of Chemical and Biomolecular Engineering, University of South Alabama, Mobile, AL 36688, USA; 2 Department of Systems Engineering, University of South Alabama, Mobile, AL 36688, USA; 3 Department of Pharmacology, University of South Alabama, Mobile, AL 36688, USA; 4 Center for Lung Biology, University of South Alabama, Mobile, AL 36688, USA

**Keywords:** endoscopy, system architecture, system lifecycle, hyperspectral, subsystem trends, model-based systems engineering (MBSE), future endoscope

## Abstract

Systems engineering captures the desires and needs of the customer to conceptualize a system from the overall goal down to the small details prior to any physical development. While many systems projects tend to be large and complicated (i.e., cloud-based infrastructure, long-term space travel shuttles, missile defense systems), systems engineering can also be applied to smaller, complex systems. Here, the system of interest is the endoscope, a standard biomedical screening device used in laparoscopic surgery, screening of upper and lower gastrointestinal tracts, and inspection of the upper airway. Often, endoscopic inspection is used to identify pre-cancerous and cancerous tissues, and hence, a requirement for endoscopic systems is the ability to provide images with high contrast between areas of normal tissue and neoplasia (early-stage abnormal tissue growth). For this manuscript, the endoscope was reviewed for all the technological advancements thus far to theorize what the next version of the system could be in order to provide improved detection capabilities. Endoscopic technology was decomposed into categories, using systems architecture and systems thinking, to visualize the improvements throughout the system’s lifetime from the original to current state-of-the-art. Results from this review were used to identify trends in subsystems and components to estimate the theoretical performance maxima for different subsystems as well as areas for further development. The subsystem analysis indicated that future endoscope systems will focus on more complex imaging and higher computational requirements that will provide improved contrast in order to have higher accuracy in optical diagnoses of early, abnormal tissue growth.

## 1. Introduction

Systems engineering (SE) is a holistic engineering skillset and mindset structured to decompose large, complex systems down to nuts and bolts and ones and zeros prior to “breaking ground” on design and fabrication. The documentation produced from SE procedures is comparable to an instruction manual that traces those bolts and bytes into components, assays, and subsystems culminating to the final system. Simultaneously, the “manual” provides parameters and verification metrics that should be met at every level of decomposition to ensure the end product’s output is productive, safe, and correct for all stakeholders involved. A note of importance in the SE process is that the documentation produced should maintain a level of abstraction to allow for creative, inventive, and cost-effective design when producing physical aspects of the system. For example, a future smart city system needs to dynamically transmit data to the populous of autonomous vehicles on the street at X Mb/s. The requirement does not dictate that it should be Bluetooth or 5G link; it could be a new method of data transfer.

Systems engineering also tracks a system throughout its lifecycle, from conception to retirement and disposal. For this manuscript, we coin a new term called “system lifetime”, which considers many different lifecycles of a system throughout history, in other words, many generations of development of a system. Noteworthy models for lifecycle processes are the waterfall and spiral model [1]. These models are primarily used for software systems, but are also exemplary models for the iterative process of producing a physical system to meet the requirements set forth in the conceptual phase. These models have been beneficial to the structuring of this work considering the lifetime of a system. Considering the waterfall or spiral in a three-dimensional space with lengths of time between new waterfalls or spirals provided a unique concept to organize the many lifecycles of system development over the course of the system lifetime. Furthermore, Hossain and colleagues have recently detailed a review of systems thinking topics through a bibliometric analysis to highlight past trends and determine current gaps in knowledge of systems thinking [2], and we made use of some of the approaches presented in this review to analyze the development of endoscope systems and potential future directions. While SE is beneficial for new, large, complicated systems such as smart cities, green energy infrastructure, and digital medical recording techniques, it can also be utilized to review and optimize smaller, complex, existing technology such as the endoscope.

Endoscopy is a medical screening process by which internal (normally hollow) organs are imaged by the insertion of a scope with illumination and imaging capabilities. Through visualization, clinicians can optically diagnose infection, inflammation, or lesion growth and resect portions of tissue for pathological diagnostics. There are four major endoscopic techniques widely used today: white light endoscopy (WLE), narrow-band imaging (NBI), Fujifilm flexible spectral imaging color enhancement (FICE), and virtual endoscopy (VE). WLE is the gold standard technique used for decades to capture a typical RGB (red–green–blue) image providing reflectance-based images of the luminal wall [3–5]. NBI illuminates body cavities with blue and green light to harness the light absorption of the vasculature at these wavelengths providing additional contrast to the image [6,7]. FICE is a post-acquisition process that divides the RGB image into the respective three colors and digitally alters wavelengths to enhance the contrast [8,9]. VE uses coherent tomography scanning (CT scan) or magnetic resonance imaging (MRI) to render a 2D or 3D model of the hollow cavities traditionally imaged with an endoscope [10]. These techniques are detailed further for their strengths and weaknesses in Section 3. Pathologies of the gastrointestinal (GI) tract can at times be difficult to differentiate from the surrounding mucosa [4,11–13]. While current techniques provide several complementary modes for visualizing internal body cavities, the contrast and definition between healthy and afflicted tissue is limited, especially in early-stage cancer. The limited contrast between some cancers and the surrounding mucosa can have downstream consequences on detection accuracy and patient outcomes, for example in colorectal cancer, which is the third-ranking cancer in the United States for incidence and mortality rates [14–17]. Neoplasia (abnormal cellular growth) can be difficult to observe within the mucosal lining. If missed, neoplasia can become invasive and malignant (cancerous); in essence, we have let a cellular vehicle run a red light without getting ticketed. Tumor growth (1) can approximately double in volume annually [18,19], and (2) the standard of care for interval routine endoscopic screenings is 5 years [5]. Further, (3) a missed colorectal tumor could approximate a minimum 32× volume increase (assuming a constant exponential growth rate) before being detected at a subsequent colonoscopy. Therefore, it is important to develop improved technologies that provide high contrast and the ability to visualize neoplasia or early-stage cancer when viewing the hollow organs endoscopically.

Here, we aimed to present a historical review of the origins and development of the endoscope similar to the approach described in Julius H. Comroe, Jr.’s Retrospectroscope [20]. Comroe’s work reviewed technologies that were created secondary to the original intent or a culmination of separate inventions. The objectives of this review are to: (1) highlight major milestones throughout the lifetime of the endoscopic system (in the following section), (2) track changes or improvements of different components or subsystems, and (3) theorize what future endoscope systems will involve based on the review and existing (potentially unrelated) technologies. To achieve these objectives, a review of the endoscope was performed, system-level requirements and architecture were constructed at predetermined milestones, system elements were observed throughout the review to acknowledge areas for future improvement, and a Pugh matrix scoring method was constructed to assess potential technology improvement areas for future endoscopes. A unique aspect of this work is a first-of-its-kind system engineering analysis for the endoscope by deconstructing major aspects of the system using model-based systems engineering (MBSE). The review then allows for speculation of what the future of the technology will be using preliminary decision-making tools. SE diagrams and graphical representations of component upgrades (i.e., light source evolving from a candle to high-powered LEDs) were used to visualize the evolution of the endoscope system. The architecture was developed in a system modeling software (Astah) and for the brevity of the review section, key elements of the system in each milestone are condensed into a figure for respective subsections. This provided insight into which aspects of endoscopy have been fully optimized and which can still be enhanced. Areas of improvement lead to the final section theorizing the future directions of endoscope technologies. Endoscopy has been reviewed multiple times [21–25]; however, this article combines the historical perspective with a SE structure. System architecture highlights the traceability of a component throughout the endoscopic system and throughout its lifetime in the system. The overall goal of this historical perspective is to provide a system-level understanding of the endoscope that will serve as the basis for developing the next generation of endoscopic technology for enhanced contrast of tissue components, especially neoplastic growth.

## 2. Historical Overview

The historical overview of the endoscopic system is sectioned into 5 milestones that marked significant leaps in functionality: origin, electricity and the light bulb, fiber optics, imaging and video, and finally the current version(s). Several other achievements are noted within these milestones to provide a comprehensive history of the endoscope. At each milestone, the systems architecture is updated to reflect the major changes to the system.

### 2.1. The Origin Story

**Figure 1.**
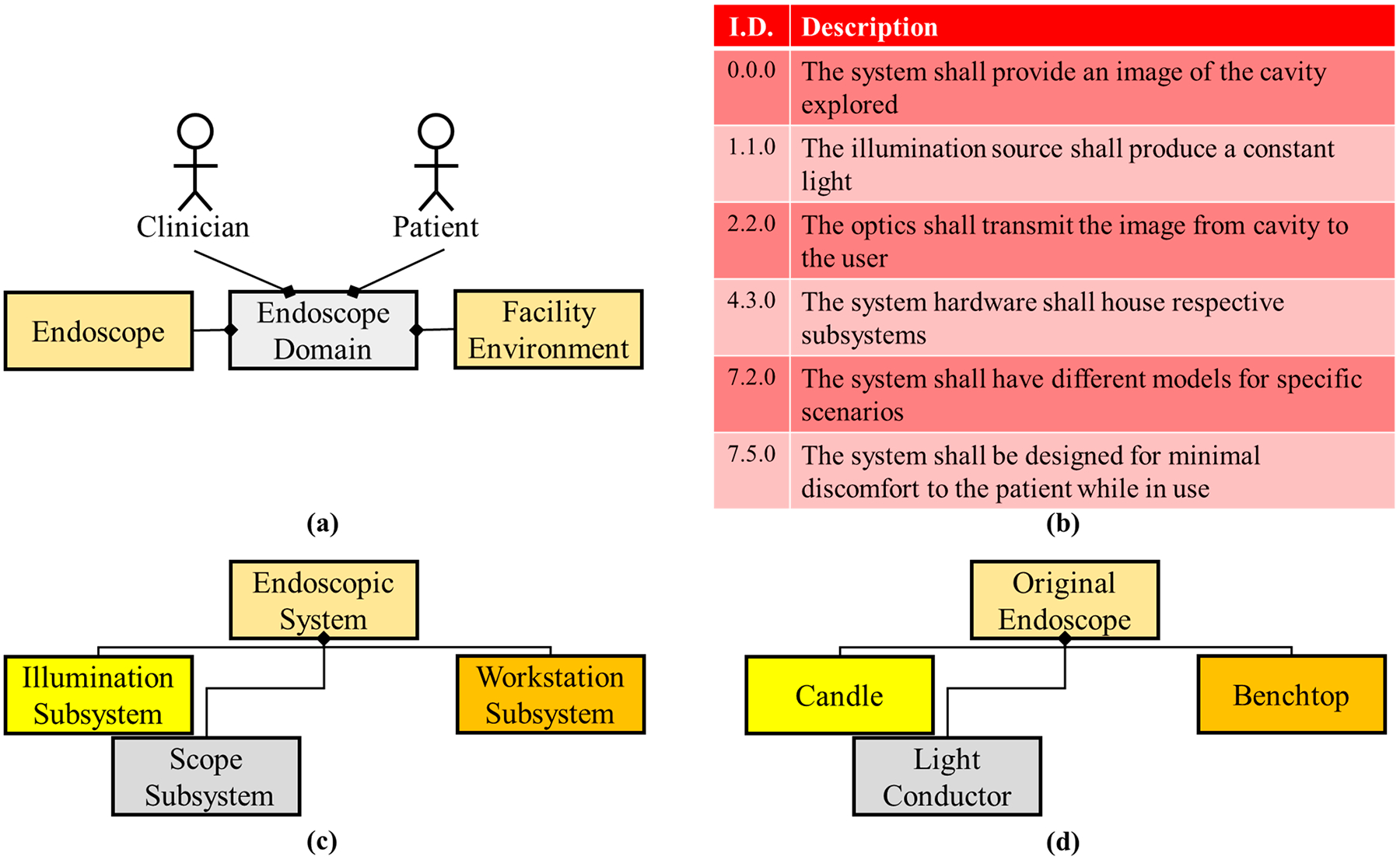
Condensed system architecture for the original (Lichtleiter) endoscope. (**a**) Domain diagram representing all actors and systems involved in the endoscopic domain. The original domain only concerned two actors (clinician/user and patient), the endoscope system and the environmental system. (**b**) A sub-selection of the requirements necessary for the original design extracted from Bozzini’s documents which state some basic requirements that have carried through to the current version of the system. (**c**) Logical architecture of the system and a decomposition into subsystems. The system was broken down into 3 subsystems. (**d**) Physical architecture of the endoscope system highlighting major components within each subsystem (i.e., the candle and the light conducting hardware).

Philipp Bozzini, a German physician, is credited as inventing the first endoscope (the Lichtleiter—light conductor) in 1806 [26,27]. Bozzini’s manuscript states that there was a desire to be able to visualize the internal hollow cavities and organs such as the bladder, rectum, and pharynx. Hence, by this period in time, medicine had developed sufficiently that physicians knew that optimal care and treatment could come from visualizing the internal organs of the body. Bozzini had defined requirements needed for the original endoscope and the resulting system was impressive because most of the metrics still apply today (Figure 1) [26,28].

In the design of the first endoscope, Bozzini implemented concepts that are still in use today (in a modern form). Illumination was held constant by placing the candle on a spring within the housing so that as the candle burned the spring would keep the flame in the same position. The Lichtleiter insertion tubing was designed to expand the naturally compressed hollow organs. Additionally, there were various-sized insertion attachments to minimize discomfort for the respective human orifices. The original endoscope only scratched the surface of visualizing the internal organs with the short depth of insertion and low illumination of the candle, but this was an amazing foundation system because the main function and concepts have been applicable throughout this system’s lifetime, even for the technology we know today.

Antonin Desmoreaux improved the endoscopic system by replacing the candle with a gasogene (alcohol and turpentine) lamp for illumination (1853). He also coined the term endoscopy for the first time. This was the best and the brightest option for the time period; however, these light sources were only practical for illuminating internal tissues at short depths.

### 2.2. A Bright Idea

**Figure 2.**
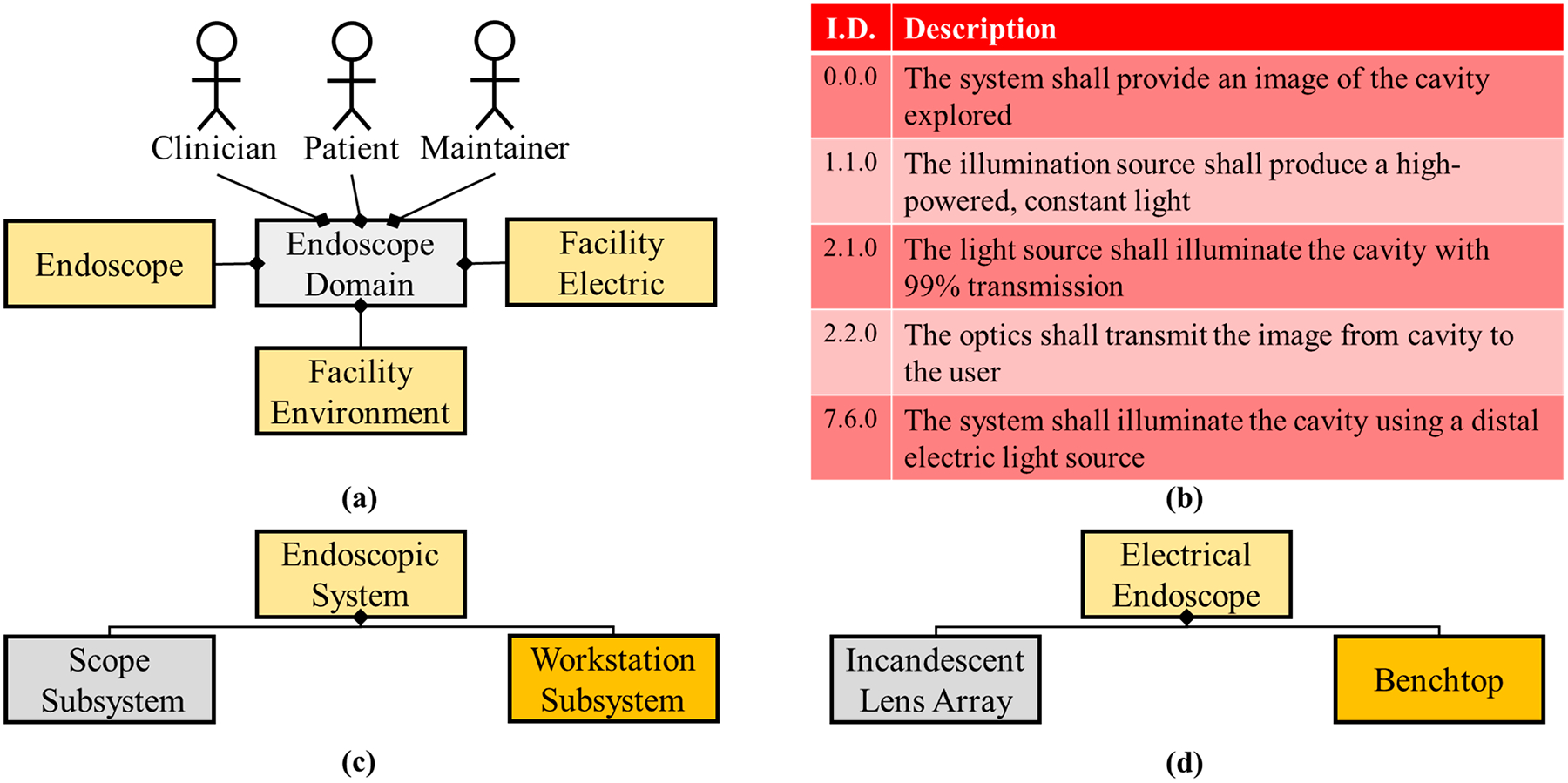
System architecture for the endoscope integrating the electric light bulb. (**a**) A maintainer actor has been added to the domain diagram due to a more complex system with components that degrade. The facility electric system was included in the domain as well due to the light bulb integration (**b**) The requirements are updated in addition to the requirements presented in Figure 1b. (**c**) Logical architecture is peculiar in this generation of endoscopes because an illumination subsystem does not exist, and hot illumination (miniature light bulb on the distal tip) is within the scope subsystem. (**d**) Physical architecture included the light bulb (incandescent) and Nitze’s lens array.

Illumination changed when society began to harness electricity via electric cells (Alessandro Volta, 1800) [29] and electric generators (Michael Faraday, 1831) [30]. This spurred the invention of the light bulb and long-term electric lighting. Humphry Davy illuminated a charcoal strip wired to a battery, the first “light bulb” (1809) [31]. A platinum filament illuminated in a vacuum tube by Warren de la Rue (1840) [32] presented an expensive, long-lasting light source. In 1867, Julius Bruck used this same technique in an endoscope system as the first hot illumination source (light source at the distal end of the scope) [33]. Henry Woodward and Matthew Evans patented the light bulb in 1875 [34]. Joseph Swan enclosed the light bulb in a glass bulb (1878) [35,36]. The original light bulb patent was bought by Thomas Edison (1879) and updated to the commercially available incandescent light bulb [37–39]. This technology was miniaturized and implemented into endoscopy by David Newman and Maximilian Nitze eight years later [40]. This chain of events made the illumination source for endoscopy much brighter but the system architecture more straightforward (Figure 2).

Ironically, the original requirements (Figure 1b) still apply at this milestone although an electric light bulb has been added. Updated requirements were implemented due to the use of a “hot illumination” electric light source and accounting for illumination transmission through the system. Other updates to the endoscopic system during this time period included increased length of scope and advanced optics to transmit the image back to the user. Adolph Kussmaul visualized the upper GI with a 0.47 m long tube, a gas lamp (before the implementation of electric lighting), and the help of a sword-swallower in 1868 (the first esophagoscope) [41]. After the inclusion of filament lighting, Max Nitze implemented a lens array [42] (design for telescopes) into a longer rigid endoscope to enhance the image (1879). However, the enhancements were nominal due to miniature light bulbs that produced inadequate illumination and the large air gaps in Nitze’s lens array.

### 2.3. “Savings” When You (Fiber) Bundle

**Figure 3.**
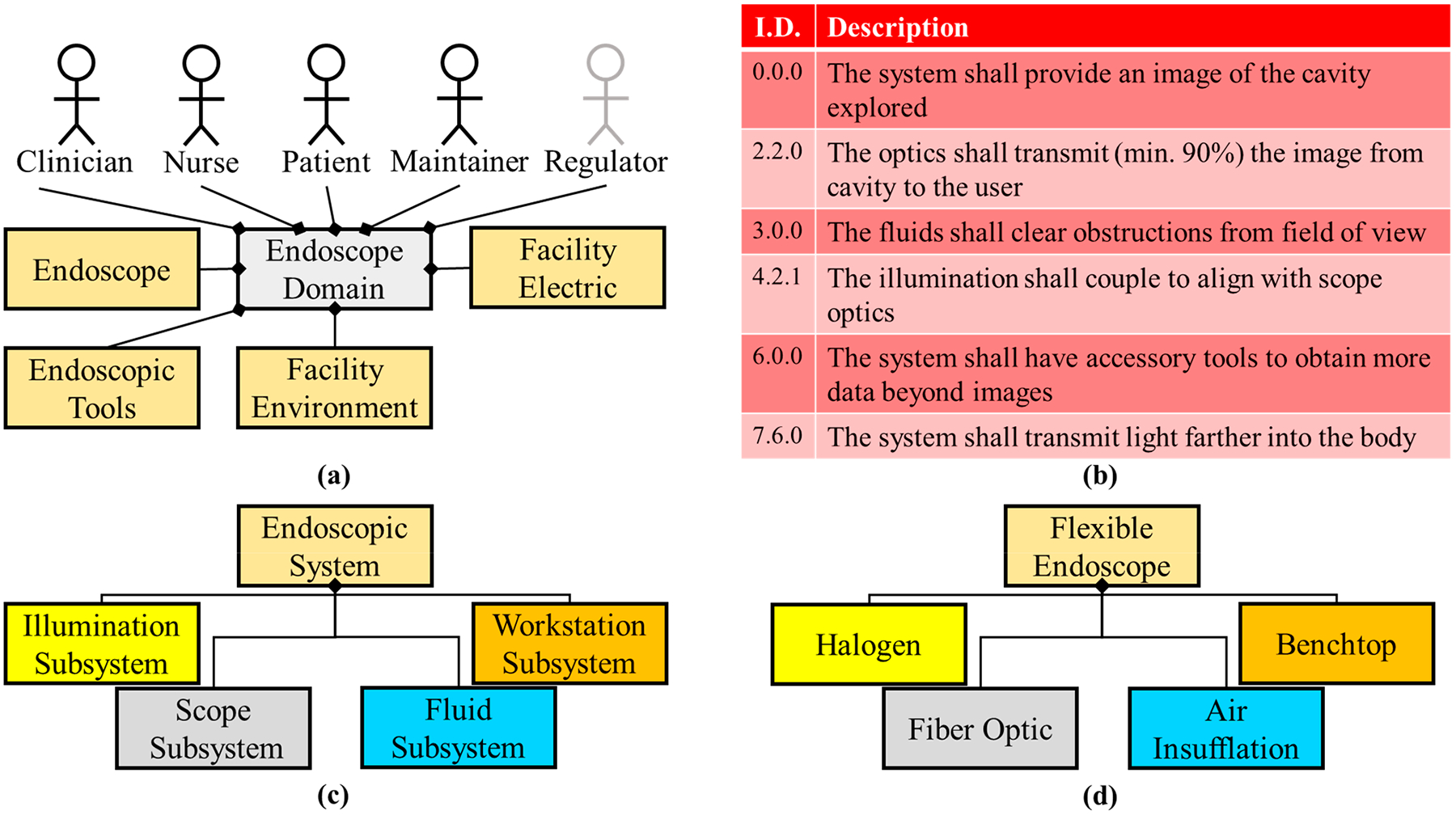
The endoscope milestone involving significant optical updates. (**a**) The domain diagram for the optical milestone includes the additional actors of the regulator (the FDA was established in 1906) and the nurse (nursing programs were commonplace by 1950), as compared to the domain diagram of the electrical milestone (Figure 2a). The regulator is a passive stakeholder denoted by increased transparency. Around this time endoscopic tools were introduced during procedures and are indicated in the domain diagram as a secondary system. (**b**) A summary of the requirements for this milestone. These requirements are updated or in addition to the requirements presented in Figure 1b and Figure 2b. (**c**) The illumination and the fluid subsystem were added to the logical diagram due to cold illumination and insufflation. (**d**) Physical components of the system include halogen bulbs, the fiber optic bundle, and air insufflation.

From 1900 through to 1950, the advancement of endoscopic techniques was minimal—a proverbial dark age—until Harold Hopkins made several significant contributions to the fields of optics that would further endoscope capabilities [43,44]. First, he created the zoom lens (1948) as a general optics assembly that would later be integral in endoscopy. In 1959, Hopkins created a rod-lens system that was an optical inversion of Nitze’s lens array because Hopkin’s design created “air lenses” from the small gaps between the glass rods. The rod-lens array was designed to minimize light loss via refraction, resulting in a ninefold increase in illumination transmission over Nitze’s design. Additionally, seven years earlier (1952), Fourstier, Bladu, and Valmier made the light source of an endoscope external again (or cold illumination) due to the heat generated by distal (hot) illumination started by Bruck and Nitze [24]. This was accomplished by transmitting light down a quartz rod in the rigid scope. At this point, if a photon were personified, it would run a relay race from the light source through optics to the internal organs, and then reflect back through separate optics, resulting in an image for the user. Due to longer light paths, it was imperative that transmission was at an all-time high. This is where Hopkins’ third endoscopic achievement comes into play: the fiber bundle. Originally developed by Heinrich Lamm, the fiber bundle transmitted light from one end of these flexible silica fibers to the other [22,24]. However, Lamm was ahead of his time because the fiber’s utility was wasted until 1954 when Hopkins, with Narinder Kapany, applied the technique for illumination in endoscopy. They developed incoherent (fiber orientation irrelevant) and coherent (fiber orientation accounted for) bundles to transmit illumination and the image, respectively. This was the era of the flexible endoscope. The orientation of the fibers and therefore the image matter because around this same time photography was becoming instrumental in the medical field. The architecture for this milestone is visualized in Figure 3. During this time period, the Food and Drug Administration (FDA) was created in 1906, marking the inclusion of regulatory oversight in biomedical equipment [45]. In addition, within this century, nursing, nursing education, and the need for increased medically-trained staff were apparent [46]. Hence, a regulator and nurse stakeholder are now considered in the endoscope system domain diagram (Figure 3a). Another notable development was the inclusion of air irrigation to expand (insufflate) a body cavity so as to obtain a larger field of view [47]. Initially developed as a hand pump attachment (similar to a blood pressure cuff), this technique was later translated to a mechanical pump to provide automatic continuous insufflation. Therefore, a new subsystem (fluid) was added to the logical and physical architectures (Figure 3c,d, respectively).

### 2.4. Smile, You’re on Camera

The first endoscopic image was acquired by Nepomuk Czermak in 1860, of his own larynx [48]. Theodor Stein developed cameras that were lighter and smaller in 1873 for imaging the larynx. Max Nitze created the first endocamera in 1894, among his other notable endoscopic achievements. Paper film was produced in 1885 and celluloid in 1888 by George Eastman [49]. Color photography was theorized by James Maxwell (1861) [50], commercialized (with limited practicality) by Frederic Ives (1890) [51] and John Joly (1894) [52], and prized by Gabriel Lippmann (1908) [53]. Gastroenterologist N. Henning reported the first color endoscopic photograph in 1938 [21]. Photographic documentation for endoscopy became the status quo in 1954 marking a milestone in the screening process of endoscopy [48].

Early moving pictures notably began with devices such as the Praxinoscope and Phènakiscope in the 19th century [54]. Thomas Edison and William Dickson made another noteworthy contribution with the invention of the Kinètographe camera that filmed a video on a film reel (1891) [55]. The Lumière brothers (Auguste and Louis) popularized video documentation in 1895 with the Cinèmatographe, the first camera projector, and hosted the first public film [56]. Auguste Lumière acquired the first medical film that same year of a military doctor treating three patients in the barracks. However, video film was not used in endoscopic procedures until the 1950s, with the first televised bronchoscopy and recorded laparoscopy [57].

**Figure 4.**
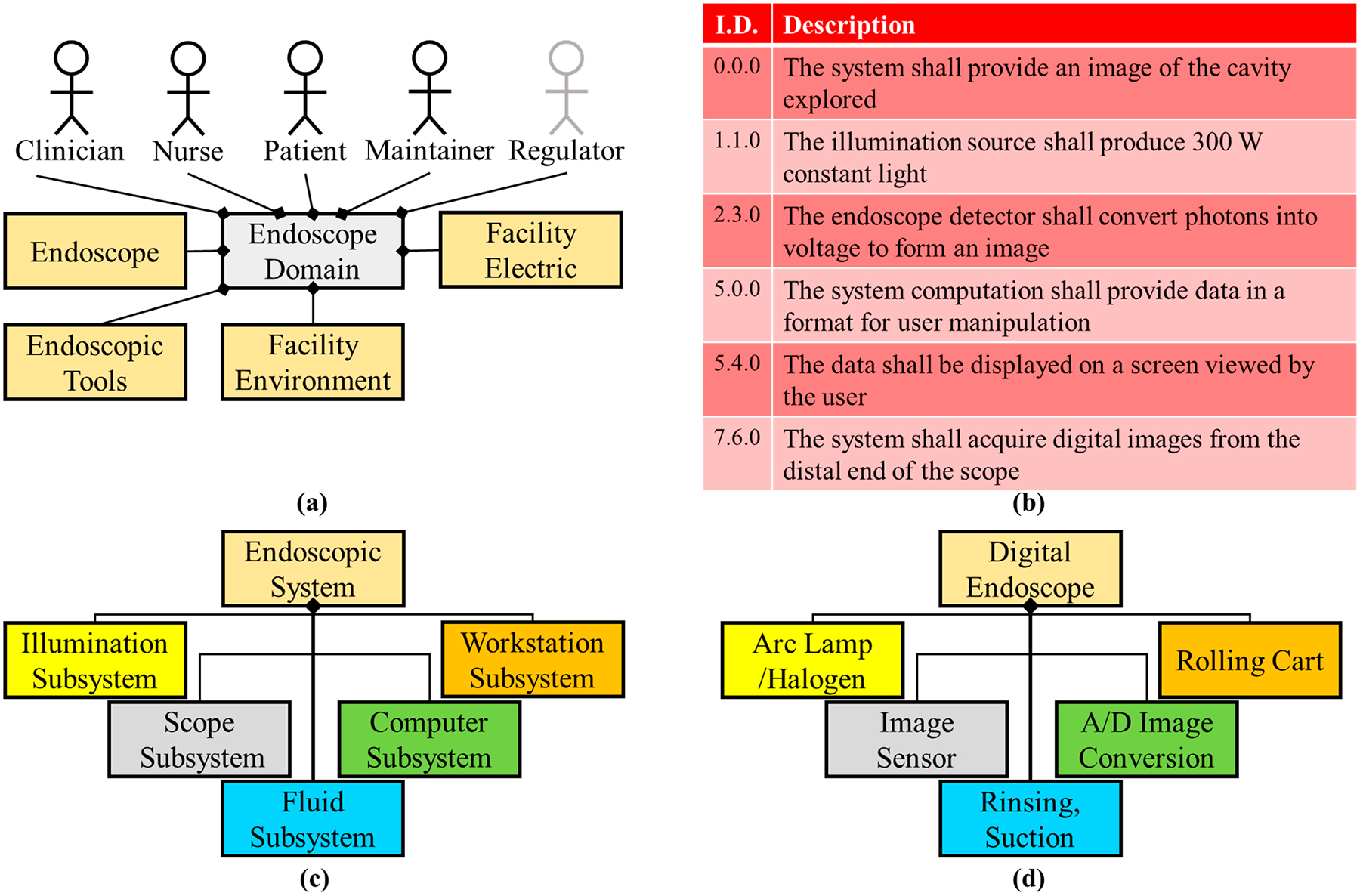
Digital imaging endoscope system architecture. (**a**) The domain diagram remains the same for this milestone when compared to the domain of Figure 3a. (**b**) The requirements assessment reflects the high-powered light sources of this era and digital imaging. (**c**) A computer subsystem was added to the logical diagram of the previous milestone (Figure 3c) due to digital imaging. (**d**) Arc lamps, CCD cameras, rinsing, and digital imaging conversion are physical component examples of this architecture.

Imaging and video acquisition was further advanced in 1969 with the invention of the charged coupled device (CCD) from Boyle and Smith at AT&T Bell Labs (additionally the complementary metal oxide semiconductor—CMOS from NASA in 1992) [58,59]. The CCD, originally developed for solid state data transfer, became a noteworthy component for digital photography. Integrated circuitry continued to improve (Moore’s Law) creating smaller pixels, more densely packed pixel areas, and therefore smaller image sensors [60–63]. The resulting component was small, cheap to produce, with low power consumption, and continually upgraded image quality. Interestingly, the combination of the camera phone in 2000 [64] and the exponential popularity of Apple’s iPhones (beginning in 2007) [65] created the driving factor for image sensor improvements and manufacturing. While the chip-on-tip image sensor was implemented for laparoscopic surgery in the 1980s [66], the image improvements for endoscopy can be correlated to the desire to improve image quality in cellular devices. Digital imaging for endoscopy, specifically laparoscopy, improved visualizing body cavities, optical diagnoses, and surgical procedures. As compared to standard open surgical techniques, laparoscopic surgery allowed for smaller incisions, reduced recovery times, and improved patient outcomes, especially for common procedures such as hernia repair, gallbladder removal, and appendectomy. The system architecture overview for the imaging milestone is shown in Figure 4.

## 3. Presenting, the Endoscope

**Figure 5.**
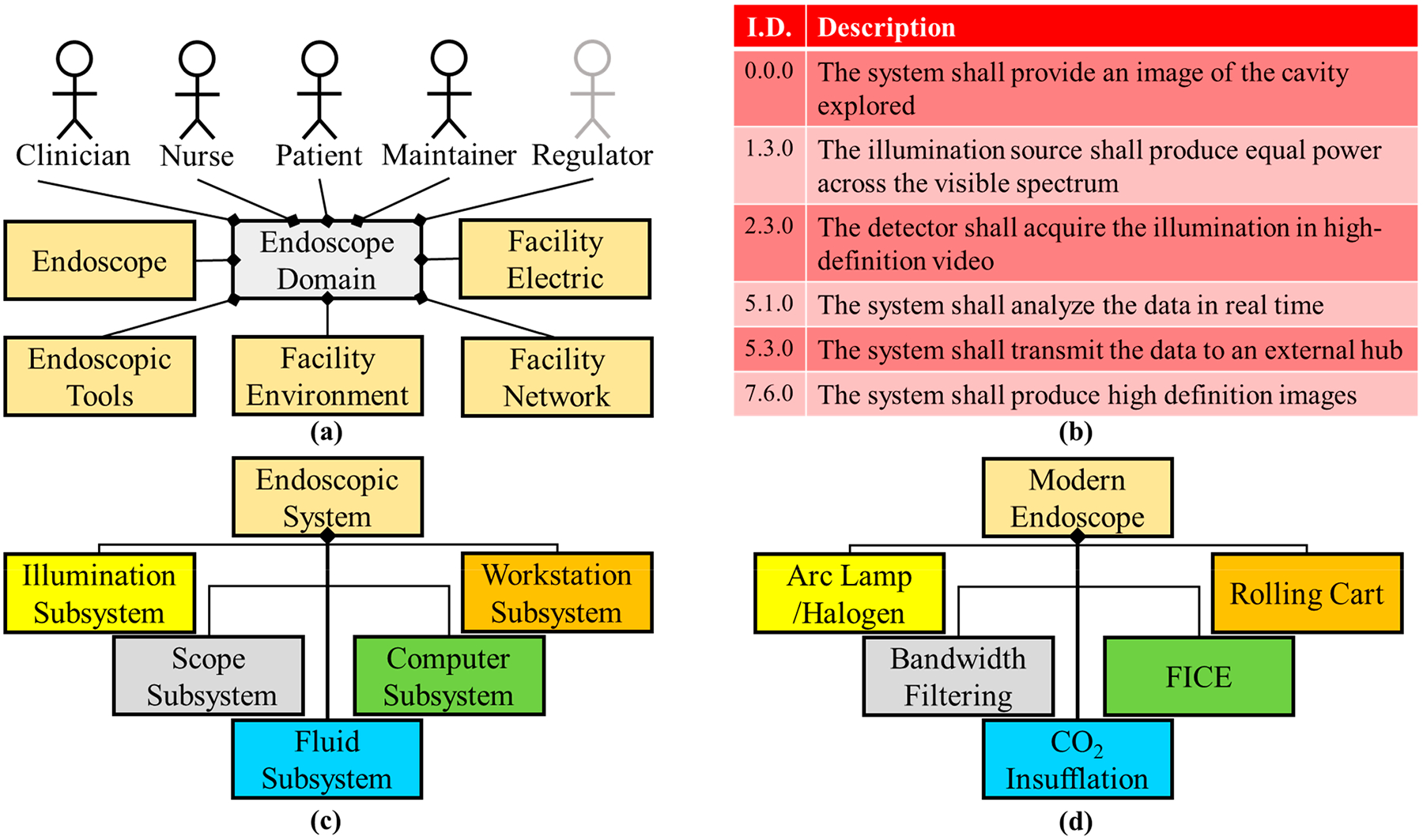
Current endoscope condensed system architecture. (**a**) The facility network external system was added to the domain diagram of the current system, compared to that of the imaging milestone domain (Figure 4a), because of the use of the internet and cloud storage. (**b**) High-definition and spectral aspects are stated in the requirements for the current system. (**c**) The logical diagram remains the same as the previous milestone (Figure 4c). (**d**) Physical components and software are bandwidth filters and FICE software for this architecture milestone.

Current state-of-the-art endoscope systems utilize a combination of broadband light sources (xenon arc lamps, halogens, or LEDs), bandwidth filtering, and digital analyses to produce WLE (gold standard), NBI, or FICE. One limitation of WLE is that small and subtle changes within the lumen may not generate sufficient contrast to be detected. In addition, abnormal, irritated, inflamed, or neoplastic tissue may appear very similar to normal tissue, resulting in difficulty determining potential areas of risk, especially for patients with underlying inflammatory conditions. To improve detection sensitivity and specificity, a range of correlating factors are often considered, such as: irregular mucosal patterns, condensed vasculature, and definitive redness. Introduced briefly before, NBI and FICE are two complementary modalities to WLE that can provide enhanced contrast of tissue structures between mucosa and lesions. NBI filters utilize narrow spectral bands in the blue and green regions to illuminate the tissue, harnessing the absorption of blood at those wavelengths and creating an image that contrasts vasculature as brown [6]. Condensed vasculature has been associated with lesional tissue due to its invasive, nutrient-draining nature. FICE is an image algorithm that uses the RGB image acquired through normal screenings and processes individual color channels into unique wavelengths (within the respective color range) that accentuates tissue differences greater than the original colored image [8]. This method has defined mucosal irregularities and tissue irritation more effectively than traditional WLE due to post-imaging processing. These techniques are included in aspects of the current architecture (Figure 5).

Another modern endoscopic screening technique is virtual endoscopy (VE) or computed tomography (CT) endoscopy [10,67]. Using CT creates a volumetric image of the entire colon or tracheobronchial tree with a noninvasive technique. VE is a beneficial screening for patients with occlusions prohibiting traditional WLE, providing a complete image of the respective body cavity and the best option for older patients or patients who are contraindicated from standard endoscopic procedures. CT data provides cross-sectional views of the organs and three-dimensional reconstruction to create a virtual scan that resembles a WLE procedure.

Another alternative to WLE is capsule endoscopy [68,69]. Capsule endoscopy is more invasive than VE, but still requires less hospital procedure time than standard WLE colonoscopy. An endoscopic camera and illumination source in capsule form is ingested and transmits video feed to wearable receiver for clinicians to view post ingestion. Current models are intuitive to their location in the gastrointestinal tract with automated data acquisition rates depending on the rate of capsule movement (data acquisition would decrease in the stomach and if the capsule slowed or stopped) and have two wide-angle cameras to ensure a full view of the colon. The fact that the capsule primarily images the small and large intestines highlights that capsule endoscopy is currently utilized primarily for colonoscopy imaging and the optimal scenario to image or view the small intestine.

Modern endoscopic techniques are a definite advancement from Bozzini’s original endoscope. Current state-of-the-art endoscopes are focused on contrasting and detecting minute tissue changes to diagnose early, screening processes that are minimally invasive, and providing as much information (the “big picture”) to benefit the patient. In the previous section, the history of the endoscope was detailed and the system upgrades were traced. Next, individual subsystems of the endoscope system were analyzed to view the trends through the endoscope lifetime and discuss where components or subsystems are currently optimal and can be improved or upgraded.

### 3.1. What’s Trending

**Figure 6.**
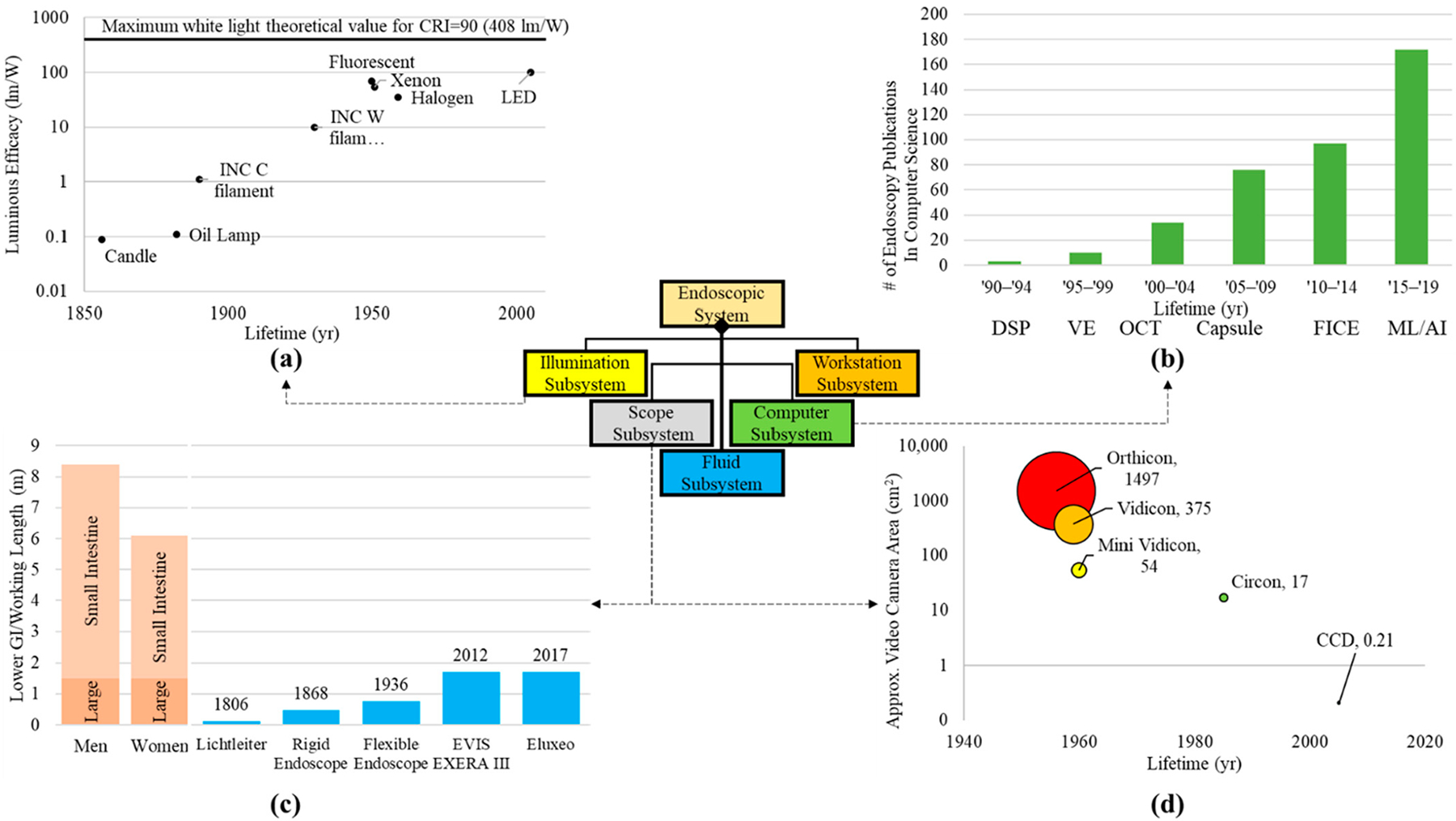
Trends for components within subsystems. (**a**) Light sources in the illumination subsystem presenting the luminous efficacy from the candle to arc lamps and LEDs. Illumination data were extracted from Azevedo et al.’s paper on solid state lighting [70]. (**b**) Advancements in computational aspects of endoscope systems were visualized by plotting the increase in endoscope-related publications within the computer science field (publication search for “endoscopy” in the computer science category per quinquennium—Source: http://www.scopus.com, accessed on 28 July 2022) [75]. Some examples of imaging processing technologies that were found include: digital signal processing (DSP), virtual endoscopy (VE), optical coherence tomography (OCT), capsule endoscopy Capsule), Fujinon’s flexible spectral imaging color enhancement (FICE), and machine learning (ML) [76]. (**c**) Optical pathway (working length) [22,28,41] for the scope subsystem showing the depth the endoscope has traversed throughout the milestones compared to the length of human body intestinal tract [77]. (**d**) Approximate camera/detector area for various cameras (both film and digital) throughout imaging in endoscopy. Camera and detector areas were assumed from dimensions given in literature [22,57,78–80].

Systematic decomposition of previous and existing endoscopic systems highlights the improvements of subsystems and elements within the system, as well as areas for potential upgrades. Here, the current milestone logical architecture is highlighted and the trends in technology are shown for particular subsystems (Figure 6). A primary advancement is the illumination source from candles to incandescent bulbs to LEDs (Figure 6a). Light sources have shown a substantial improvement in luminosity, decreased power consumption, and increased component longevity. Some literature theorizes that illumination technology is reaching maximum potential in white light luminous efficacy [70].

With regard to the computational subsystem, we examined the trend in the literature of endoscope-related computer science publications. We searched the Scopus database for publications in the field of computer science using the keywords “gastroenterology” or “endoscopy” and a date range of 1990–2019, divided into five-year increments (Figure 6b). Results indicated that the last two decades have shown a 50X increase in the number of publications fitting these parameters. Examples of the increased computational demand of such components begin with the image sensor digital signal processor (DSP) to convert photons to digital signals [71] and expand to three-dimensional (VE and optical coherence tomography—OCT) [72] and wireless or self-contained (capsule endoscopy) or enhanced channel contrast (FICE).

The optical light path which carries illumination to the patient cavity and the image back to the user or imager has also been optimized through the years (Figure 6c). The Lichtleiter with a length of ~10 cm increased to the current colonoscopes with a length over 1.5 m, capable of spanning the entire large intestine and a portion of the small intestine. These depths would not be possible if fiber optics were not introduced, creating flexible endoscopes. Furthermore, a smaller fiber optic bundle and overall endoscopic diameter allowed for the development of smaller systems such as bronchoscopes, cystoscopes, laparoscopes, and ureteroscopes for lungs, bladder, small surgical openings, and ureter, respectively [73]. Flexible endoscopy has not extended the working length further into the small intestine due to the tortuous and compact nature of the organ [74]. Maneuvering a flexible scope through the small intestine could perforate the mucosal lining or damage the fiber optics of the endoscope. Furthermore, the amount of articulation and force that could be applied to the endoscope tip decreases with increased length and depth into the lower GI. Smaller endoscopes are limited in illumination and detection due to size constraints. Therefore, the output of these smaller systems has lower spatial resolution and potentially lower contrast between normal and abnormal tissue.

Creating physical records of endoscopic screenings is one of the most important aspects of the field today. The transition from clinician hand-drawn images to film photography lessened the workload and provided an objective image to support diagnosis. Ironically, cameras continued to improve by creating clearer pictures while becoming smaller components within the system (Figure 6d). Film-based cameras were large additions to system on the proximal end. Now, digital image sensors are miniaturized on the distal end of scopes providing real-time, high-definition images and video.

### 3.2. Endoscopy: The Next Generation

**Figure 7.**
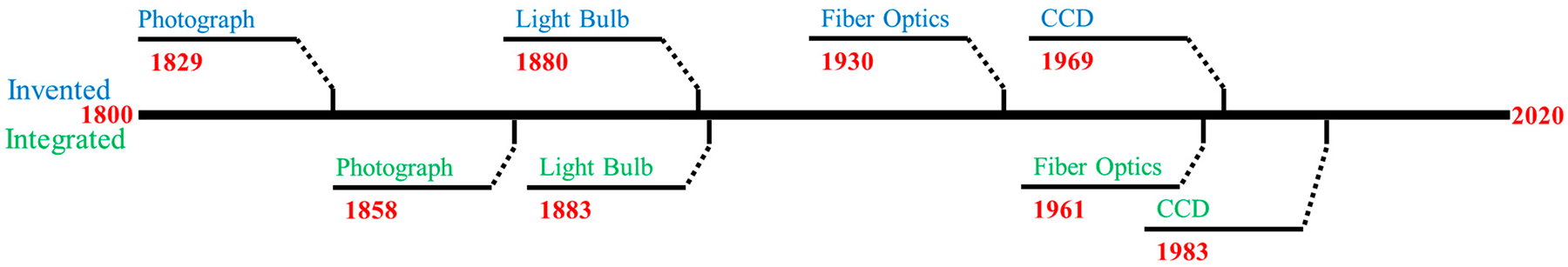
Timeline comparing technology invention dates to the time it was integrated into endoscopy. Blue text denotes invention and green for integration.

Using systems engineering architecture as a tool for review, we can exhaustively survey the needs of the range of subsystems and components, as well as environmental constraints and current technologies. We can predict which technologies may need to evolve and what a next generation endoscope would provide. Based on Figure 6, illumination is at a current maximum, digital sensors can accommodate any endoscope diameter with the caveat of limited resolution for smaller sensor sizes, the working length of the scope cannot get any longer due to highly condensed and tortuous nature of the small intestine and there is a high interest in the computational capabilities of the endoscopic technologies. Reviewing past inventions that were implemented into endoscopy, Figure 7 shows the importance of looking at off-the-shelf components and technology.

**Figure 8.**
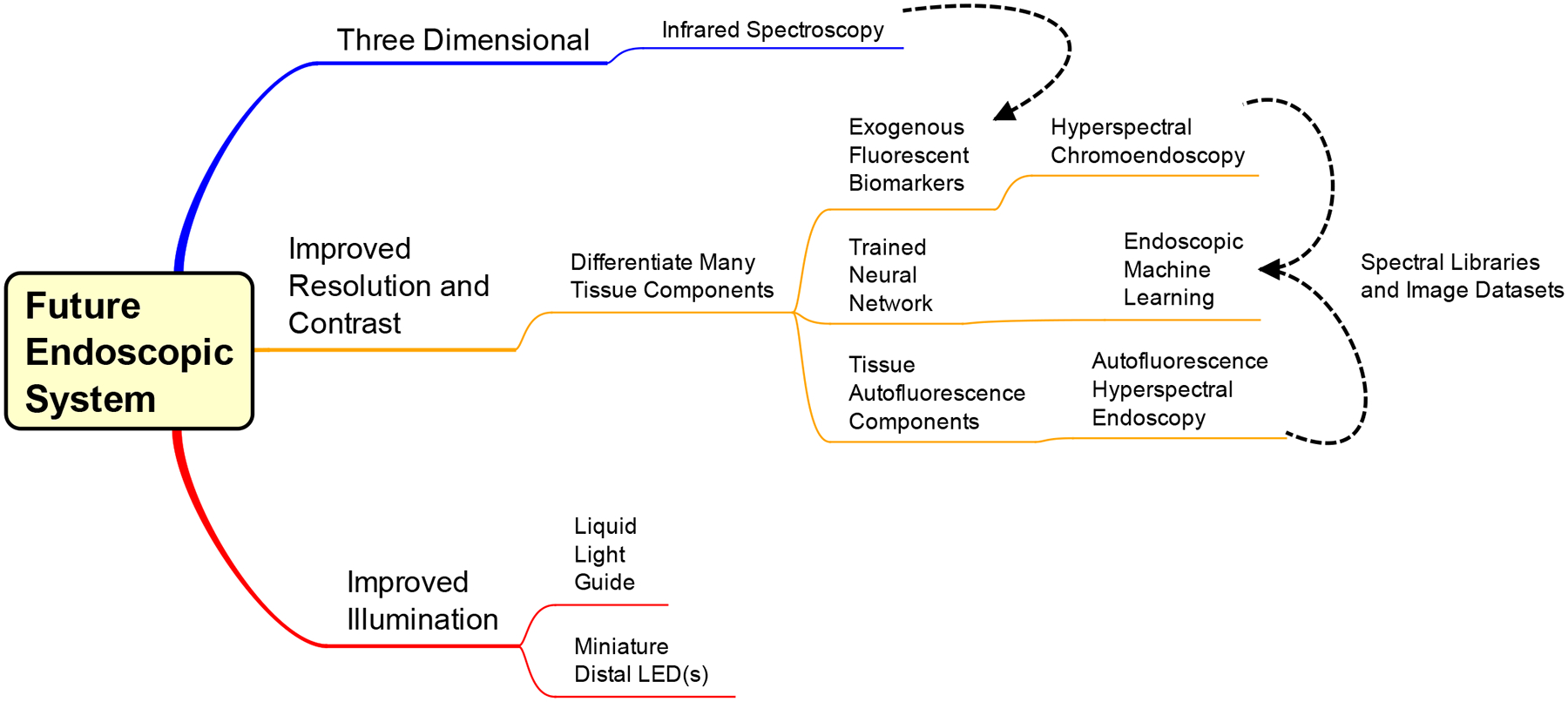
Mind map for design of a next-generation endoscopic system. The middle and top pathways are considerations of techniques to implement, and the bottom pathway is component upgrades of the current endoscope system. Dashed lines convey interconnectivity between topics: Infrared analysis could implement with exogenous fluorescent biomarkers and hyperspectral chromoendoscopy and autofluorescence hyperspectral endoscopy could be analyzed and displayed via machine learning algorithms.

The endoscopic community was quick (3 years) to integrate incandescent light bulbs to the endoscope design, but it was over 30 years between invention and implementation of the fiber optics, creating the flexible endoscope. Therefore, a technology might already exist with the potential to benefit the output of endoscopy. For the scope of this manuscript, we reference the previous upgrades and potential gaps in the system while acknowledging the technologies that exist outside the field of endoscopy. To begin justification for these possible upgrades, a mind map was constructed to outline the avenues (Figure 8).

The mind map has two parts: technology for implementation (top two pathways) and components to improve current systems (bottom pathway). Components for increased illumination throughput include miniature LEDs at the distal end of the scope and/or a liquid light guide instead of fiber bundles. When digital camera sensors became small enough, they were integrated at the distal end of endoscopes, reducing transmission losses by eliminating the secondary (imaging) light guide. If white light LEDs or three respective RGB LEDs were small enough to replace the illumination fiber optic area of the distal tip, then transmission losses would be further reduced. A major factor in creating a new hot illumination is the potential for heat dissipation and mitigating damage to the patient cavity. Another option is exchanging illumination fiber bundles for liquid light guides (LLG) to increase the throughput of the endoscope. An LLG has no void fraction, a higher numerical aperture, and a higher acceptance angle compared to fiber optic bundles accepting more illumination proximally, minimal internal refraction, and a diffuse illumination at the distal end (potentially illuminating a larger area). A limitation to using LLGs would be a slightly smaller spectral transmission range (normally 200–600 or 400–2000 nm) than that of a fiber optic bundle with broader transmittance (200–2000 nm).

Another option is infrared spectroscopy for additional information to WLE. Infrared wavelengths are longer and penetrate deeper than visible wavelengths. Therefore, there is potential for image information within deeper layers of tissue beyond the mucosa. A benefit of extended optical penetration depth could be identifying density variations in the tissue beyond the mucosal wall [81–83]. Similar to the vasculature, higher density could correlate to lesion growth, and this could enable early detection. Infrared illumination and imaging also has the potential to discriminate neoplasia from inflammation [84]. The limitation here is the need for a separate detector and illumination to outfit the infrared technology necessary to produce visible images for the user.

The mind map topics which combine new and old technologies to enhance contrast the images produced are detailed below. Here, they are labeled as: endoscopic machine learning, autofluorescence hyperspectral endoscopy, and hyperspectral chromoendoscopy. Machine learning has become an integral part of many fields, especially in applications that produce large datasets. Machine learning (ML), if implemented on a computational platform capable of real-time operation, could provide automated cues to clinicians that would aid in identifying potential abnormalities. ML outputs could be false-colored or overlayed in some other form with traditional WLE, NBI, AFI, or CE image data such that the cues are visible during a standard endoscopic technique. The requirements for ML in endoscopy need to focus on computation functioning in real-time. Hence, computational capabilities would have to be sufficiently developed to allow real-time classification and false coloring or superposition of classification results with traditional endoscopic procedures in order for this approach to be viable.

The specificity of AFI endoscopy techniques could be improved through enhanced contrast created by several endogenous fluorophores (native autofluorescence) in human tissue [85–87]. An optimal way to excite these autofluorescent biomarkers is hyperspectral imaging (particularly spectral scanning). Hyperspectral imaging generates complex image data, hypercubes, in which two dimensions represent spatial data and the third dimension represents spectroscopic data. Spectral hypercubes can be analyzed to estimate the contributions of different molecules, such as autofluorescent molecules, and these signatures can be false-colored and overlayed to generate added contrast in endoscopy images. However, autofluorescence is an inherently low signal, so the spectral illumination has to be powerful enough to provide sufficient excitation and emission signals for the detector. Current autofluorescence imaging in endoscopy typically highlights one or two endogenous molecules with one or two excitation sources [88–92]. Minimal excitation sources allow for longer acquisition and higher signal while maintaining video rates. For hyperspectral autofluorescence imaging of 5 or more endogenous molecules (assuming notable unique molecular and spectral contributions), shorter acquisition is required for the video rate, lowering the excitation signal. However, the excitation overlap could compound the excitation signal for each fluorescent biomarker. Most hyperspectral setups of this nature come with a trade-off between spatial resolution, acquisition rates, and spectral sampling. We would expect that future endoscopic system will mitigate this trade-off in order to maintain the requirements of high definition and video rate imaging, while providing hyperspectral imaging capabilities.

The requirements discussed above for autofluorescence hyperspectral endoscopy would also apply for hyperspectral chromoendoscopy to be a viable addition to the endoscopic system. In this case, exogenous fluorophores (fluorescent dyes and stains) could be used to identify certain components, tissue types, or proteins, creating a list of biomarkers to image. This fluorescence mixture could be administered during bowel preparation or during the procedure, as is performed in traditional chromoendoscopy. Hyperspectral imaging, as described above, could then be performed to allow the identification of each of the many fluorescent labels. An additional benefit to exogenous fluorophores is that they produce greater emission signaling than autofluorescence. There are also exogenous fluorophores in the near-infrared range to expand components stained and increase the contrast [93,94]. Importantly, both of these hyperspectral techniques can provide new or complimentary data for machine learning scenarios to automatically identify and flag suspicious regions for further investigation.

To understand the potential technologies that could be incorporated into a next-generation endoscope, a Pugh matrix (Table 1) was constructed. The Pugh matrix is a system engineering tool that can be used to evaluate the importance and potential impact of each endoscope technology when considering a range of parameters. Modern endoscopic procedures are included as well for reference. Scoring parameters for the Pugh matrix were determined by first considering the patient (safety, invasiveness, and comfort), then prioritizing clinician training and technology implementation (i.e., operational training and implementation cost), and finally considering the additional image data and information that could be produced (new image data and contrast). Scoring (described below) was conducted by a panel of six gastroenterologists from the University of South Alabama Division of Gastroenterology. Each category (column) within the technology row was averaged among the n = 6 responses and the standard deviation was calculated. The matrix did not include any weighted metrics and was primarily used to compare new or potential technologies to the gold standard of WLE (labeled as Current Endoscopy) and to consider which technology could represent a future next-generation endoscope. The metrics were graded as follows: Safety—How safe would this procedure be? 5 = most, 1 = least. Invasive—How invasive would this procedure be? 5 = highly, 1 = minimally. Patient Comfort 5 = comfort, 1 = discomfort. Operational Training—How much operational training would be required? 5 = extensive, 1 = minimal. Example Image Training—How much training with example images is needed? 5 = extensive, 1 = minimal. Implementation—How easy would the technology be implemented? 5 = challenging, 1 = easy. Cost to Implement—How much would this technology cost to integrate? High = $$$ = 5, Low = $ = 1. Additional Image Data—How much additional image information would be produced? 5 = substantial, 1 = minimal. High Contrast—Would this technique produce a higher contrast than WLE? 5 = substantial, 1 = minimal. The final column of the matrix totals the scores for comparison (the values are in bold to highlight the overall results of the table). For this work, Invasive, Operational Training, Training Image Data, Implementation, and Cost were all subtracted from four, so the total was the summation of the positive connotations for each technology (i.e., the “inverse” of the aforementioned metrics were considered for the total—Invasive: five translated to a one for noninvasive).

The results of this Pugh matrix, based on the criteria selected, show that any new version of the system comes with some drawbacks, mainly the time to make operational (i.e., clinical trials, FDA approval, clinical acceptance). The technological trend of the future is big data and with that comes the bottleneck of analyses and results selection that are useful for the end product. All options were comparable in safety measures and patient comfort as physical procedures remain similar to WLE. Additional training requirements were anticipated with more advanced image data and analysis technologies (i.e., hyperspectral options). However, operational training should be comparable to WLE with the exception of some software interface changes. Interestingly, the (hardware and/or software) implementation of these technologies should be a streamlined process. These alternatives do not require redesigns, but component or subsystem exchanges that should minimize design costs and fabrication challenges. The cost of implementation would be high due to training (both user training and training data) and changing subsystems (i.e., illumination and scope subsystems). Current endoscopic systems scored the highest according to the matrix, but the long-term potential and in-depth data (Additional Data column of Table 1) provided by the alternatives make the initial drawbacks or cost worth the transition. The trade-off is noticeable when comparing the hyperspectral options to WLE. The hyperspectral technologies would need advanced training (especially example imagery for users and training data for neural networks, if applicable) but are anticipated to provide increased information and increased contrast for identifying suspect lesions (as seen in the Additional Data and Contrast columns). Integrating a neural network or artificial intelligence into endoscopy also has a trade-off, as the additional information or contrast is also accompanied by a more complex data type that the clinician may have to interpret, if not sufficiently processed and summarized. Infrared imaging scored the lowest on the matrix due to the unknown factors of how the technology would integrate with an endoscope platform and how best to present the data to clinicians. Infrared imaging would require the most design-intensive change to implement a widefield technique infrared imager at the distal end of an endoscope. Regardless, the next generation of endoscopy will likely involve some aspect of machine learning and a technological advancement to improve contrast between normal and lesional tissue. Future system architectures will probably see a large increase in complexity for computing and illumination subsystems.

## 4. Conclusions

The aim of this work was to provide a historical review of the endoscope system using MBSE architecture. To our knowledge, observing the endoscope throughout the many lifecycles of development (which we defined here as the “system lifetime”) is a first-of-its-kind review for both the endoscope community and the MBSE community. From the review, the trends and changes to the technology were traced to determine where future iterations of the system are trending. This work contained three main objectives. The first objective was to present key milestones of the endoscope through systems architecture. The review indicated the systems architecture at each milestone and provided system traceability to track the changes throughout the system’s lifetime. The second objective was to track the changes and trends in components and subsystems of endoscopy. The traceability of the first objective highlighted the key components for which trends in development could be quantified resulting in a visualization of improvements over the system lifetime and areas for further research. The third objective was to theorize future technologies for endoscopy. The results indicate that complex and/or computationally-focused image technologies are important areas for the development of future endoscope systems. The history of the endoscopic system has a fascinating timeline from Bozzini’s brilliant inception, to sword-swallower patients, to the integration of several improved illumination and camera technologies over the last 50 years. Endoscopy provides numerous ways the clinician can screen the internal tissues benefitting patient care. This field also revolutionized the way surgeries are conducted and how medical data are produced via imagery. The system architecture produced here has been an invaluable perspective for tracking the changes and additions throughout the last 200 years, as well as highlighting areas with potential for further optimization.

The future of endoscopy will require new imaging techniques that provide increased information and contrast. Based on this review, we predict that imaging will become more complex, or the endoscope system will provide opportunities to combine complementary imaging techniques so as to produce data that provide increased contrast and accuracy of optical diagnoses. Additionally, we anticipate that there will be an increase in computational requirements to accommodate more advanced imaging techniques. One primary computing requirement will be increased in silico computing capabilities to analyze image data in real-time. It is also important to consider that implementing new technologies will come with trade-offs, including the need for higher computing power, more training, or more complex devices. However, the increase in data to provide increased contrast and detection accuracy will outweigh the aforementioned trade-offs. Whatever the future of endoscopy presents, these trade-offs will be worth it long-term to provide superior care for the patient, more knowledge of the human body, and properties/attributes of disease progression.

Standard systems engineering analysis excels when the voice of the customer is involved and the use cases and requirements are defined. However, the historical aspect of this work makes involving past customers and users impossible. The requirements and use cases presented here are a mixture of literature and decomposition from the current system. We strived to keep the architecture unbiased, but we know that with assumptions there comes some bias elements. This is also true for the speculation of future endoscopic systems. For the scope of this work, we are providing our perspective based on knowledge of the current system and similar technologies. The hope is that this architecture created can be used as a foundation for others in the fields (both endoscopy or biomedical imaging and systems engineering) to glean new ideas and find additional trends to bolster the next generation system even more. Similar to sensational skyscrapers, the future endoscopic system will need a great set of blueprints (architecture) and inputs from people (customers) in several fields and clinical settings. The goal of endoscopy remains the same, to provide an image of the cavity explored (to benefit the patient’s long-term health). Hopefully, exploring the past endoscopes will establish new endoscopes with higher quality, more in-depth images.

## Figures and Tables

**Table 1.** Pugh matrix ranking alternative technologies compared to the gold standard (WLE). Metrics for scoring include safety, training, implementation, and added information. The scores are totaled for comparison.

	Safety	Invasive	Patient Comfort	Operational Training	Example Image Training	Implementation *	Cost to Implement	Additional Image Data	Higher Contrast	Total
Current Endoscopy	4.3 ± 0.7	3.0 ± 1.6	4.0 ± 1.2	4.2 ± 0.7	3.7 ± 0.7	3.3 ± 1.4	2.8 ± 1.5	3.5 ± 1.4	3.0 ± 1.3	**17.8**
Virtual Endoscopy	4.7 ± 0.5	2.5 ± 1.5	4.5 ± 0.5	4.0 ± 0.8	4.2 ± 0.7	3.5 ± 0.8	3.5 ± 1.4	3.3 ± 1.1	3.0 ± 1.5	**17.8**
Capsule Endoscopy	4.2 ± 0.7	2.8 ± 1.2	4.0 ± 0.6	3.8 ± 0.7	4.2 ± 0.7	3.2 ± 1.3	3.5 ± 0.8	3.3 ± 1.1	2.8 ± 1.3	**16.8**
Infrared Imaging Endoscopy	3.7 ± 0.9	3.3 ± 1.2	4.0 ± 0.8	4.2 ± 0.4	4.2 ± 0.7	4.0 ± 0.8	3.8 ± 0.7	3.8 ± 0.7	3.2 ± 1.2	**15.2**
Autofluorescence Hyperspectral Endoscopy	4.0 ± 0.8	3.3 ± 1.2	4.0 ± 0.8	4.3 ± 0.7	4.7 ± 0.5	4.5 ± 0.8	3.7 ± 1.5	4.2 ± 0.9	4.0 ± 1.4	**15.7**
Hyperspectral Chromoendoscopy	3.8 ± 0.7	3.3 ± 1.2	4.2 ± 0.9	4.5 ± 0.8	4.7 ± 0.5	4.3 ± 0.7	3.8 ± 1.5	4.5 ± 0.8	4.3 ± 1.1	**16.2**
Neural Network Endoscopy	4.2 ± 0.9	3.5 ± 1.4	4.3 ± 0.9	4.7 ± 0.5	4.2 ± 0.9	4.2 ± 1.1	3.7 ± 1.9	4.2 ± 1.2	4.2 ± 1.2	**16.7**

Scoring: 5 = highly, most likely or effectively, 1 = minimally, least likely or effectively, * 5 = challenging, 1 = easy.
